# Data on stable isotopic composition of δ^18^O and δ^15^N in nitrate in groundwater, and δ^15^N in solid matter in the Varaždin area, NW Croatia

**DOI:** 10.1016/j.dib.2022.108686

**Published:** 2022-10-21

**Authors:** Igor Karlović, Tamara Marković, Andrew Smith, Tjaša Kanduč

**Affiliations:** a Croatian Geological Survey, Milana Sachsa 2, 10000 Zagreb, Croatia; b National Environmental Isotope Facility, British Geological Survey, Nicker Hill, Keyworth, Nottingham NG12 5GG, UK; c Department of Environmental Sciences, Jožef Stefan Institute, Jamova Cesta 39, SI-1000 Ljubljana, Slovenia

**Keywords:** Nitrogen, Stable isotopes, Nitrate sources, Groundwater contamination, Varaždin aquifer

## Abstract

The Varaždin aquifer is the only source of drinking water for inhabitants of the Varaždin County. In the last decades, groundwater contamination with nitrate has become an increasing problem. Therefore, there is a need to define the origin of nitrate as the first step in groundwater remediation. The data in this article consist of δ^18^O and δ^15^N values in nitrate in groundwater, and δ^15^N in solid matter. Groundwater was sampled in the period from April 2018 to December 2019 at 10 different sites by pumping the wells, and directly in the gravel pit in Šijanec. Representative solid samples of plants, soil, manure, and synthetic fertilizers were collected from arable land in two field campaigns (July and October 2019). The presented dataset can be used as a baseline for identification of nitrate sources in groundwater and possible nitrate attenuation processes. The data is related to the research article “Tracking the nitrogen cycle in a vulnerable alluvial system using a multi proxy approach: case study Varaždin alluvial aquifer, Croatia.” [1].

**Specifications Table**

**Table:** 

Subject	Environmental Science
Specific subject area	Stable isotope analysis
Type of data	Table Figure
How data were acquired	Groundwater and solid samples collected in the field. δ^18^O-NO_3_ analysis was undertaken on an TC pyrolysis elemental analyser (EA) coupled to a Thermo Fisher Delta XL Isotope Ratio Mass Spectrometer (IRMS). δ^15^N-NO_3_ analysis was undertaken on a Flash elemental analyser (EA) coupled to a Thermo Fisher Delta XL IRMS. δ^15^N analysis in solid samples was measured with Isoprime 100 IRMS coupled to an Elementar PyroCube preparation module.
Data format	Raw Analyzed
Parameters for data collection	The data refers to groundwater (δ^18^O and δ^15^N in NO_3_) and solid matter (δ^15^N).
Description of data collection	Fieldwork was conducted to collect 60 groundwater samples for δ^18^O and δ^15^N stable isotope analyses in nitrate. Preliminary sampling campaign was conducted in April 2018, followed by nine monthly sampling campaigns from April 2019 to December 2019. In total 60 samples were collected from 10 different sampling sites by pumping the wells, with exception of gravel pit in Šijanec where water was taken both by water pump and directly by submerging the bottle below the water level. Isotope ratios of δ^18^O and δ^15^N in nitrate were measured with IRMS technology in the National Environmental Isotope Facility at the British Geological Survey. Solid matter samples were collected from arable land in two sampling campaigns (July and October 2019). Total 17 solid samples were collected from agricultural fields, including plant, soil, manure, and synthetic fertilizer. Isotope ratios expressed as δ^15^N were measured with IRMS technology in Department of Environmental Sciences of the Jožef Stefan Institute.
Data source location	Institution: Croatian Geological Survey City/Town/Region: Varaždin Country: Croatia Latitude and longitude for collected samples/data: Varaždin area 46.32627 °N, 16.243802 °E
Data accessibility	Repository name: Mendeley Data Data identification number: 10.17632/54rkwj3kdg.1 Direct URL to data: https://data.mendeley.com/datasets/54rkwj3kdg/1
Related research article	T. Marković, I. Karlović, S. Orlić, K. Kajan, A. C. Smith, Tracking the nitrogen cycle in a vulnerable alluvial system using a multi proxy approach: case study Varaždin alluvial aquifer, Croatia, Sci. Total Environ. 853 (2022) 158632. *https://doi.org/10.1016/j.scitotenv.2022.158632*

## Value of the Data

• Presented data provide a unique insight into nitrogen dynamics, enable identification of nitrate sources in groundwater and indicate possible processes that could attenuate nitrate concentrations in groundwater.
• Data can be useful for researchers dealing with the nitrogen cycle, especially hydrogeologists and geochemists working on groundwater contamination related problems. Nitrogen transformation processes are mediated by microorganisms, so the data could be interesting to microbiologists dealing with bacteria linked to the nitrogen cycle. As groundwater is an important source of drinking water, researchers involved in water quality management may also be interested in data, e.g. to formulate management strategies and specific measures to reduce nitrate contamination in groundwater.
• The dataset can be used for development of groundwater model, primarily for defining the origin of nitrate in groundwater and exploring the possibility for simulation of the retardation processes. Characteristic δ^15^N values in solid matter present a valuable data of various nitrogen sources from which nitrate in groundwater originates, i.e. δ^15^N fingerprint. The contribution of each nitrate source can be quantified using mixing models.

## 1 Objective

The presented data article is related to an original research article “Tracking the nitrogen cycle in a vulnerable alluvial system using a multi proxy approach: case study Varaždin alluvial aquifer, Croatia” published in Science of The Total Environment. The data was an important factor in establishing the source of groundwater contamination with nitrates, on the basis of which further groundwater protection measures can be taken.

## 2 Data Description

**Fig. 1:**
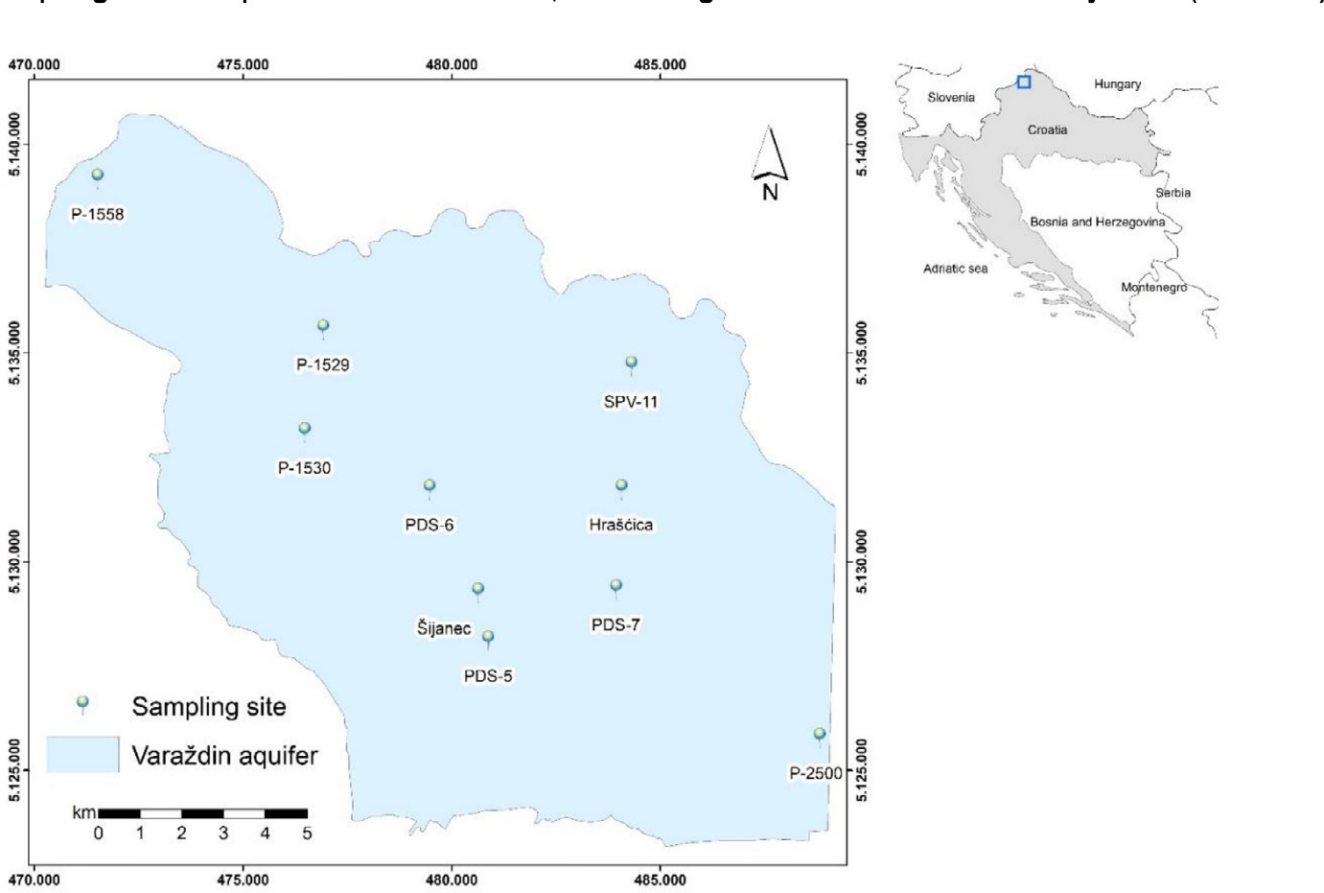
Map of the Varaždin area with location of the sampling sites.

The data presented include stable isotope ratios of δ^18^O and δ^15^N in nitrate in groundwater and δ^15^N in solid matter. The location of sampling sites in the Varaždin area are depicted in Fig. 1. in the official coordinate system of the Republic of Croatia (HTRS96/TM). The coordinates for each sampling site are presented in Table 1, according to the World Geodetic System (WGS84).

**Table 1:** Coordinates of sampling sites.

Sampling site	Description	Latitude(° N)	Longitude(° E)	Elevation (m a.s.l.)
Hrašćica	Private well	46.325158	16.293106	176.00
PDS-5	Observation well	46.292462	16.25173	178.36
P-2500	Observation well	46.271647	16.354878	167.81
PDS-7	Observation well	46.303596	16.291492	175.71
SPV-11	Observation well	46.351711	16.296098	177.69
PDS-6	Observation well	46.324993	16.233373	184.07
P-1529	Observation well	46.359419	16.200068	187.32
P-1530	Observation well	46.337192	16.194381	183.72
P-1558	Observation well	46.391673	16.129586	193.97
Šijanec	Water pump	46.303247	16.249053	179.70
Šijanec	Gravel pit	46.302756	16.24857	178.80

The dataset in XLSX format includes two tables: (i) Groundwater and (ii) Solid matter and is deposited in Mendeley Data online repository [2]. Groundwater data table presents sampling date, sample volume, NO_3_-content, δ^18^O and δ^15^N in nitrate in 60 groundwater samples distributed in 10 sampling sites. Solid matter data contains sampling date and δ^15^N values in plants, soil, manure, and synthetic fertilizers.

## 3 Experimental Design, Materials and Methods

### 3.1 δ^15^N and δ^18^O in nitrate in groundwater samples

Groundwater samples (n = 60) were collected in the field from 10 sampling locations. Preliminary sampling campaign was carried out in April 2018, followed by nine monthly sampling campaigns from April 2019 to December 2019. The groundwater was sampled by pumping from nine observation wells, except sampling site in Šijanec where sample was taken by installed water pump and by submerging the bottle below the water level in the gravel pit. Technically, the gravel pit is surface water that was formed by the excavation of aquifer material, but is presented here as groundwater because it is a groundwater discharge zone, and the water level in the pit is actually at groundwater level. At least three volumes of groundwater from each observation well were pumped out before sampling to provide a representative sample from the aquifer. Samples for nitrate content analyses were filtered through 0.45 µm membrane filters into a 50 mL HDPE plastic bottles with a tight-fitting cap, on the basis of which the volume required for isotopic analyzes of δ^15^N and δ^18^O in nitrate was determined. A successful isotope measurement requires 1 to 2 mg of NO_3_-N. The samples for nitrate content analyses were transported in a portable refrigerator and measured by ion chromatography (Dionex ICS 6000) in the Hydrochemical Laboratory of the Croatian Geological Survey immediately upon returning from the field. Samples for isotope analyses of δ^15^N and δ^18^O in nitrate in groundwater were filtered through 0.2 µm membrane filters into the HDPE 200/500/1000 mL bottles with a tight-fitting cap, transported in a portable refrigerator and frozen upon arrival at the laboratory. The frozen groundwater samples were transported to the National Environmental Isotope Facility of the British Geological Survey (UK), where isotope analyses of δ^15^N and δ^18^O in nitrate were performed.

Dissolved nitrate in groundwater samples was converted to AgNO_3_ salt according to the methods described in Chang et al. (1999) and Silva et al. (2000) [3,4]. This procedure allows the extraction of nitrate using two conditioned ion exchange columns. The appropriate volume of sample is passed through cation resin column to remove cations, followed by an anion resin column to absorb nitrate. Nitrate was eluted from the anion resin using 25 mL of HBr and neutralized with 2.5-3 g of washed Ag_2_O. The solution was filtered through a 0.2 µm polycarbonate filter to remove the AgCl, and 4 ml of 1 M BaCl_2_ was added and left overnight to precipitate non-nitrate oxygen-bearing anions. The solution was then passed through cation resin column to remove excess barium, re-neutralized with 1 g of Ag_2_O, and filtered before freezing overnight. The frozen sample was freeze-dried to obtain solid AgNO_3_, then re-dissolved in 1 mL of MilliQ water, after which the samples were centrifuged and ready for mass spectrometry. An appropriate volume of AgNO_3_ solution was pipetted into a silver capsule, freeze-dryed and wrapped for measurements of oxygen isotopes (δ^18^O-NO_3_). The analysis was performed on an TC pyrolysis elemental analyser (EA) coupled to a Thermo Fisher Delta XL Isotope Ratio Mass Spectrometer (IRMS). Before analysis, the EA-IRMS was checked for stability through a series of reference gas (CO) injections and tuned to achieve optimum sensitivity. Silver nitrate samples were introduced to the EA via a zero blank autosampler first flushed by lab grade He (99.9%). The sample was pyrolyzed at 1450 °C in the presence of glassy carbon and graphite, silver residuals are captured in a graphite crucible for disposal. The sample gas then passed onto the IRMS for isotope analysis with 0% sample dilution on CO. Samples for nitrogen isotope analysis (δ^15^N-NO_3_) were prepared by pipetting an appropriate volume of AgNO_3_ solution to a silver capsule, freeze-drying, adding 0.5 mg of sucrose, closing and wrapping in a tin capsule. The analysis was performed on a Flash elemental analyser (EA) coupled to a Thermo Fisher Delta XL IRMS. Before analysis, the EA-IRMS is checked for stability through a series of reference gas (N_2_) injections and tuned to achieve optimum sensitivity. Silver nitrate samples were introduced to the EA via zero blank autosampler first flushed by lab grade He (99.9%). The sample was combusted at 1050 °C in the presence of oxygen before passing over copper wires to reduce any residual oxygen, silver and tin residuals are captured in a glass crucible for disposal. The sample gas then passed onto the IRMS for isotope analysis with 0% sample dilution on N. Raw isotope values were produced on the Thermo software by comparing the peak area of the sample with laboratory working tank gas. Whilst this "working gas" was injected directly into the IRMS, all external reference materials were weighed into caps and pyrolyzed/combusted during the same run as the samples: IAEA-N1, IAEA-N2, and IAEA-NO_3_ for δ ^15^N-NO_3_ and USGS-32, USGS-34, USGS-35, and IAEA-NO_3_ for δ ^18^O-NO_3_. To achieve accurate measurements, raw isotope values were corrected offline for blank, drift and linearity issues before correction using a two, or three-point calibration with IAEA-NO_3_ as a check standard. All stable isotope results are reported using conventional delta (δ) notation in permil (‰) with respect to international standards: VSMOW (Vienna Standard Mean Ocean Water) for oxygen, and AIR (atmospheric N_2_) for nitrogen. The analytical precision of measurements is <1.5‰ for δ^18^O-NO_3_, and <0.3‰ for δ^15^N-NO_3_, based on within run replication of reference materials.

### 3.2 δ^15^N in solid samples

Solid matter samples (n = 17) were collected in two field sampling campaigns (July and October 2019). Samples were taken directly from arable land to represent the typical soil, plants, manure and synthetic fertilizers of the Varaždin area. Solid samples were collected into plastic bags and transported to the Geochemical Laboratory of the Croatian Geological Survey, where they were frozen, freeze-dryed, and finely ground. The prepared samples were transported to Department of Environmental Sciences of the Jožef Stefan Institute, where *δ*^15^N measurements in solids were performed.

An appropriate amount of each sample was added into tin capsules (approximately 3 to 5 mg of plants and manure, 1 mg of synthetic fertilizers, and 10 mg of soil samples) to determine the isotopic composition of nitrogen (δ^15^N). The prepared samples were measured using the Elementar PyroCube preparation module coupled to an Isoprime 100 IRMS. The accuracy of the measurements was controlled with reference materials IAEA-N1 (ammonium sulphate) with value of +0.4‰, and IAEA-310 (A1) with value of 47.2‰.

## Ethics Statement

The authors declare that the hereby presented data and data article fully comply with the Journal's policy in terms of authors’ duties, data integrity, and experimental requirements.

## CRediT authorship contribution statement

**Igor Karlović:** Investigation, Conceptualization, Methodology, Writing – original draft. **Tamara Marković:** Investigation, Data curation, Supervision, Writing – review & editing. **Andrew Smith:** Methodology, Writing – review & editing. **Tjaša Kanduč:** Methodology, Writing – review & editing.

## Declaration of Competing Interest

The authors declare that they have no known competing financial interests or personal relationships which have, or could be perceived to have, influenced the work reported in this article.

## Data Availability

Dataset on stable isotopic composition of δ18O and δ15N in nitrate in groundwater, and δ15N in solid matter in the Varaždin area, NW Croatia (Original data) (Mendeley Data).
